# Current perspectives and trends of CD39-CD73-eAdo/A2aR research in tumor microenvironment: a bibliometric analysis

**DOI:** 10.3389/fimmu.2024.1427380

**Published:** 2024-08-12

**Authors:** Tian Huang, Xiangqing Ren, Xiaolong Tang, Yuping Wang, Rui Ji, Qinghong Guo, Qian Ma, Ya Zheng, Zenan Hu, Yongning Zhou

**Affiliations:** ^1^ The First Clinical Medical College, Lanzhou University, Lanzhou, China; ^2^ Department of Gastroenterology, The First Hospital of Lanzhou University, Lanzhou, China; ^3^ Gansu Province Clinical Research Center for Digestive Diseases, The First Hospital of Lanzhou University, Lanzhou, China; ^4^ The Second Department of Gastrointestinal Surgery, Affiliated Hospital of North Sichuan Medical College, Nanchong, Sichuan, China; ^5^ The First Department of Geriatrics, Xianyang First People’s Hospital, Xianyang, China

**Keywords:** CD39, CD73, adenosine, A2aR, tumor microenvironment

## Abstract

**Background and objective:**

Extracellular adenosine (eAdo) bridges tumor metabolism and immune regulation. CD39-CD73-eAdo/A2aR axis regulates tumor microenvironment (TME) and immunotherapy response. In the era of immunotherapy, exploring the impact of the CD39-CD73-eAdo/A2aR axis on TME and developing targeted therapeutic drugs to enhance the efficacy of immunotherapy are the current research hotspots. This study summarizes and explores the research trends and hotspots of the adenosine axis in the field of TME to provide ideas for further in-depth research.

**Methods:**

Literature information was obtained from the Web of Science core collection database. The VOS viewer and the bibliometric tool based on R were used to quantify and identify cooperation information and individual influence by analyzing the detailed information of the global annual publication volume, country/region and institution distribution, article authors and co-cited authors, and journal distribution of these articles. At the same time, the distribution of author keywords and the co-occurrence of author keywords, highly cited articles, and highly co-cited references of CD39-CD73-eAdo/A2aR in the field of TME were analyzed to determine research hotspots and trends.

**Result:**

1,721 articles published in the past ten years were included in this study. Through bibliometric analysis, we found that (1) 69 countries and regions explored the effect of the CD39-CD73-eAdo/A2aR on TME, and the research was generally on the rise. Researchers in the United States dominated research in this area, with the highest total citation rate. China had the most significant number of publications. (2) Harvard University has published the most articles in this field. (3) 12,065 authors contributed to the publication of papers in this field, of which 23 published at least eight papers. STAGG J had significant academic influence, with 24 published articles and 2,776 citations. Co-cited authors can be clustered into three categories. Stagg J, Allard B, Ohta A, and Antonioli, L occupied a central position in the network. (4) 579 scholarly journals have published articles in this field. The journal FRONTIERS IN IMMUNOLOGY published the most significant number of papers, with 97 articles and a total of 2,317 citations, and the number of publications increased year by year. (5) “The ectonucleotidases CD39 and CD73: Novel checkpoint inhibitor targets” was the most frequently local cited article (163 times). The “A2A adenosine receptor protects tumors from antitumor T cells” was the most co-cited reference (224 times). (6) Through the analysis of author keywords, we found that the relationship between adenosine and immunotherapy was a core concept for many researchers in this field. Breast cancer, melanoma, colorectal cancer, ovarian cancer, glioblastoma, pancreatic cancer, hepatocellular carcinoma, and lung cancer were the most frequent cancer types in adenosine-related tumor studies. Immunotherapy, immunosuppression, immune checkpoint, and immune checkpoint inhibitors were the hot keywords in the research, reflecting the importance of the adenosine metabolic pathway in tumor immunotherapy. The keywords such as Immunogenic cell death, T cells, Sting, regulatory T cells, innate immunity, and immune infiltration demonstrated the pathways by which adenosine affected the TME. The famous author keywords in recent years have been immunotherapy, immunogenic cell death, inflammation, lung cancer, and gastric cancer.

**Conclusion:**

The effect of CD39-CD73-eAdo/A2aR on the infiltration and function of various immune cells in TME, tumor immunotherapy response, and patient prognosis has attracted the attention of researchers from many countries/regions. American scholars still dominate the research in this field, but Chinese scholars produce the most research results. The journal FRONTIERS IN IMMUNOLOGY has published the wealthiest research in the field. Stagg J was a highly influential researcher in this field. Further exploration of targeted inhibition of CD39-CD73-eAdo/A2aR alone or in combination with other immunotherapy, radiotherapy, and chemotherapy in treating various cancer types and developing effective clinical therapeutic drugs are continuous research hotspots in this field.

## 1 Introduction

Immunotherapy using antibodies against anti-CTLA-4 or anti-PD-1/L1 has revolutionized the cancer treatment paradigm (1). However, despite unprecedented responses to these therapies in some tumor types, many patients still fail to achieve clinically relevant responses in these indications, and some tumor types show resistance to immune checkpoint inhibitors (ICIs) (2).

Metabolic reprogramming is one of the critical features of tumors. Compared with normal cells, tumor cells have substantial metabolic heterogeneity and meet their needs for rapid proliferation, growth, and spreading through metabolic reprogramming. When tumor develop from precancerous lesions to local invasion, especially metastasis and treatment resistance, their metabolic phenotype and dependence will change (3). Extracellular adenosine triphosphate (eATP) and extracellular adenosine (eAdo) released into the tumor microenvironment (TME) are potent modulators of tumor cells and immune responses. DNA damage induced by certain cytotoxic drugs and radiation has been shown to trigger the release of eATP (4, 5), which is usually low (10–100 nM) in most tissues but can be elevated 1000 times in response to tissue damage, hypoxia, ischemia, or cell death (6). The accumulation of eATP is an important damage-associated molecular pattern that helps to initiate the inflammatory process and tumor antigen presentation, triggering immunogenic death of tumors, mainly by activating ion-gated channel receptors (P2XR) or selective G protein-coupled receptors (P2YR) expressed on immune cells (4, 7).

**Figure 1:**
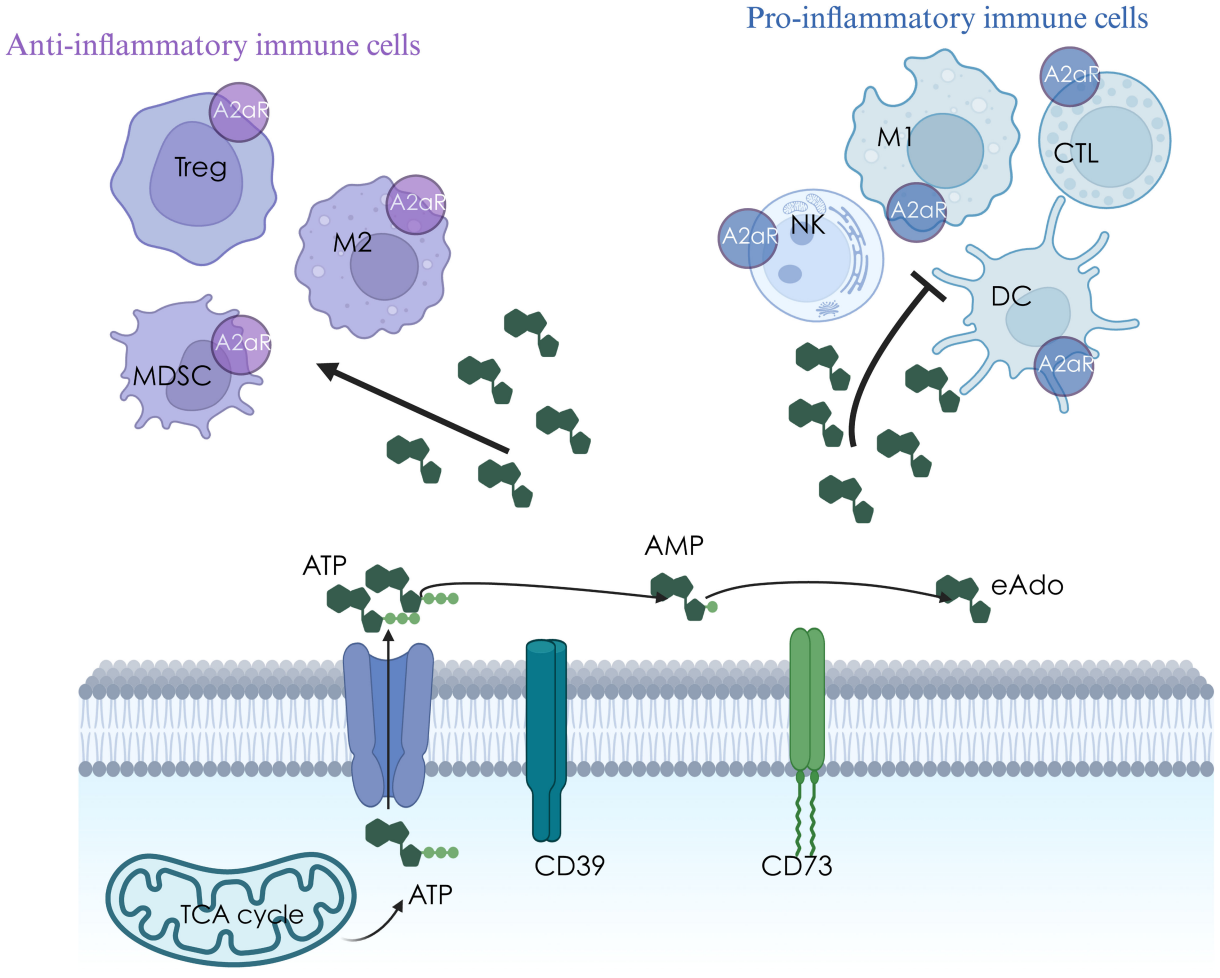
The mechanism diagram of CD39-CD73-eAdo/A2aR axis in tumor microenvironment.

eATP can also be hydrolyzed to eAdo by specialized ectonucleotidase. CD39 hydrolyzes eATP/eADP to eAMP (8), and CD73 converts eAMP to eAdo (9). CD39 and CD73 constitute vital enzymes that metabolize from pro-inflammatory eATP to immuno-suppressive eAdo. eAdo can inhibit the activation of dendritic cells, the production of Th1/Th2 cytokines, the infiltration of cytotoxic T lymphocytes (CTLs), the activation, maturation, and cytotoxicity of natural killer (NK) cells, and enhance the pro-tumor effects of regulatory T (Treg) cells, TR1 cells and tumor-associated macrophage (TAM) by binding to A2aR expressed on immune cells (**Figure 1**) (10). Cytotoxic stress and radiation can induce ectonucleotidase expression (7, 11, 12).

In addition to the classical immunosuppressive checkpoint PD-1/PD-L1, CTLA-4, the critical adenosine anabolism enzymes CD39/CD73 have also been proposed to act as immunosuppressive checkpoints, driving a shift from an eATP-induced pro-inflammatory environment to an eAdo-induced anti-inflammatory environment. The half-life of eAdo in TME is less than 10 s, and direct targeting of eAdo is impractical. Therefore, many preclinical and clinical studies have attempted to improve immunosuppression by targeting eAdo-generating metabolic enzymes (CD39 and CD73) to inhibit eAdo production or block A2aR. Targeting the hypoxia-adenosine-A2aR pathway is a cancer immunotherapy strategy to prevent suppression of antitumor T cells in the TME (13–15).

Bibliometrics is an interdisciplinary field that uses mathematical and statistical methods to analyze various forms of knowledge carriers quantitatively. Information visualization technology can intuitively display the research history, status, hotspots, and discipline development trends. In this paper, the research status and direction of CD39-CD73-eAdo/A2aR in TME were analyzed by VOS viewer and R-based bibliometric tools. This article aims to show the research trend of CD39-CD73-eAdo/A2aR in the field of TME systematically and comprehensively, find new research hotspots and topics, and provide specific references for readers to understand the research field comprehensively and deeply.

## 2 Materials and methods

### 2.1 Data collection

The Web of Science Core Collection (WoSCC) database is widely used in bibliometrics. Data is obtained from the WoSCC database from 2015 to March 22nd, 2024. The search formula is ((((((AB=(CD39)) OR AB=(CD73)) OR AB=(adenosine)) OR AB=(ENTPD1)) OR AB=(NT5E)) OR AB=(A2aR)) OR AB=(A2AR) AND ((((AB=(immune microenvironment)) OR AB=(immune)) OR AB=(microenvironment)) OR AB=(tumor microenvironment)) OR AB=(Tumor immune microenvironment) AND (((((((AB=(Tumor)) OR AB=(Neoplasm)) OR AB=(Tumors)) OR AB=(Neoplasia)) OR AB=(Cancer)) OR AB=(Cancers)) OR AB=(Malignancy)) OR AB=(Benign Neoplasm). The publication type included is “article” or “review”, and the language is “English”.

### 2.2 Data analysis

The bibliometric tool based on R (version 4.3.0) and VOS viewer (16) (version 1.6.20) are used to quantify and identify the individual impact and cooperation information by analyzing these articles’ detailed information, such as annual global publications, distribution of countries/regions and institutions, journals, authors, and co-cited authors. Meanwhile, we identify the researching hotspots and trending by analyzing the keywords, co-occurrence author keywords, and the co-cited references of CD39-CD73-eAdo/A2aR research in cancer immunity.

## 3 Results

### 3.1 The data overview in publication outputs

**Figure 2:**
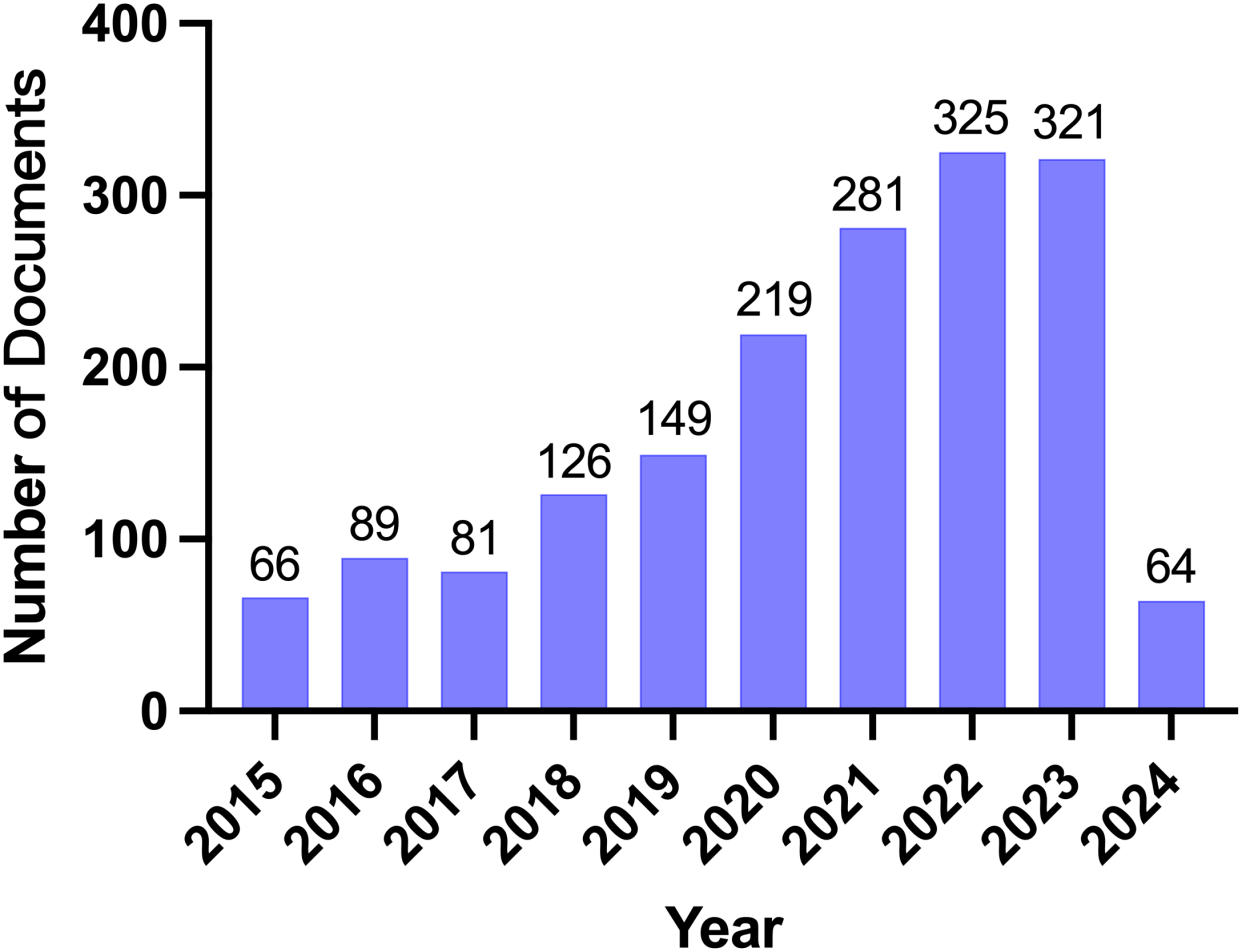
Annual publications from 2015 to 2024.

We collected the research results of eAdo synthesis key enzymes (CD39, CD37), adenosine, and A2aR in the field of TME from the Web of Science core collection database, screened the search results, and only included the articles and reviews in the past ten years on March 22nd, 2024. The basic situation of the search results is shown in **Table 1**. A total of 1,721 eligible papers are found. The number of publications in this field has increased over the past decade (**Figure 2**). From the perspective of author cooperation, more than 27.08% of the research results involve international cooperation, which reflects that this field is a topic of global concern, and there are frequent international academic exchanges and cooperation. In addition, the average number of co-authors in a single article is nine, which means that the research involved in this field is complex and requires multi-faceted knowledge and techniques. Considering the above data, we speculate that CD39-CD73-eAdo/A2aR are essential molecular markers or therapeutic targets in TME research. With the continued growth in the amount of literature and the high citation rate, this field will likely continue to expand, and research will be further in-depth, possibly discovering new treatments or drugs. The high proportion of international cooperation also indicates that there may be more international cooperation projects and transnational research teams to promote the understanding and application of CD39-CD73-eAdo/A2aR as targets in tumor immunotherapy.

**Table 1:** Data overview of papers related to CD39-CD73-eAdo-A2aR in TME from 2015 to 2024.

Description	Results
MAIN INFORMATION ABOUT DATA	
Time span	2015:2024
Sources (Journals, Books, etc)	579
Documents	1721
Document average age	3.52
Average citations per document	31.06
References	74905
DOCUMENT CONTENTS	
Keywords Plus (ID)	3767
Author’s Keywords (DE)	3323
AUTHORS	
Authors	11665
Authors of single-authored documents	28
AUTHORS COLLABORATION	
Single-authored documents	31
Co-authors per document	9.03
International co-authorships %	27.08

### 3.2 Distribution of countries/regions and institutions

**Figure 3:**
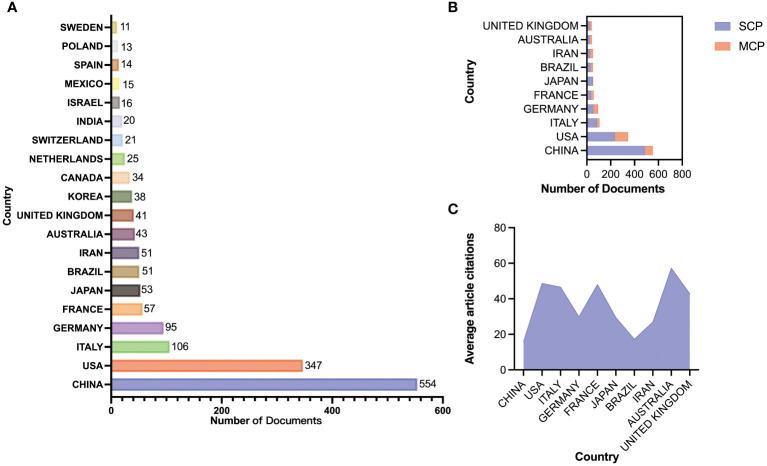
Paper publication status in various countries. **(A)** Number of publications from the top 20 countries. **(B)** Patterns of international collaboration in paper publication: Single Country Publications (SCP); Multi-country Collaboration Publications (MCP). **(C)** Average article citations of each country.

**Figure 4:**
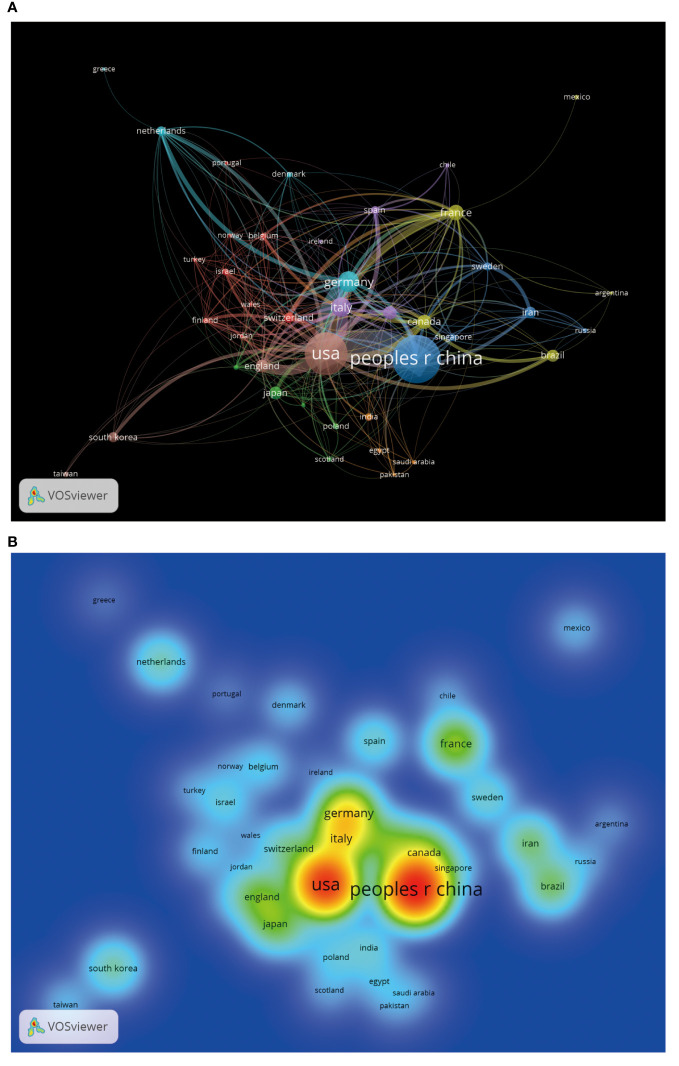
The visualization of cooperation between countries by VOSviewer. **(A)** International cooperation network diagram. The same-colored logo indicates the cluster group of countries, the thickness of the line reflects the closeness of cooperation, and the node’s size corresponds to the number of papers published by each country. **(B)** Heat map of cooperation analysis between countries. Each label represents a country, and the color on the heat map changes gradually from cool to warm, indicating low to high cooperation.

**Figure 5:**
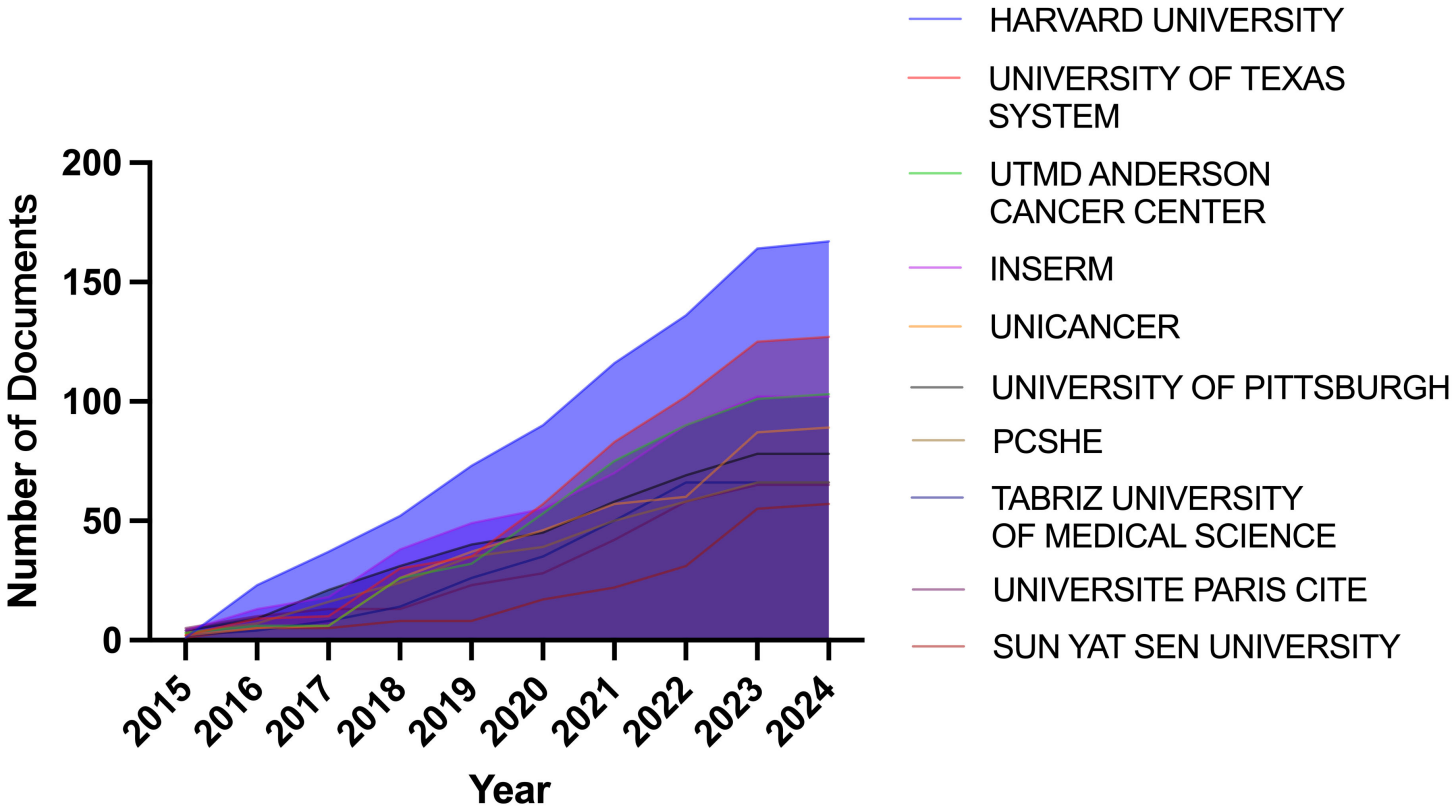
Annual publication growth trend chart of institutions.

Through the country and institutional analysis of bibliometric analysis, we can understand the research contributions of various countries and institutions in the adenosine axis and TME to navigate better and participate in this field. **Table 2** presents the details of the top 10 productive countries/regions. So far, 69 countries have published papers in this field, of which China has the most papers (554), followed by the United States (347) and Italy (106) (**Figure 3A**). The lead of China in the number of documents published by a single country (448) indicated that China has a strong research capacity and high output in this field. However, the United States leads in multi-country collaborative publications (112), stating its activity and leadership in international scientific research collaboration. The high ratio of multi-country collaborative papers in Iran, Australia, and the United Kingdom suggests that these countries are more likely to collaborate across borders in scientific research, which helps drive broader scientific progress and innovation (**Figure 3B**). The United States has the highest number of citations (16,902 times), followed by China (8,794 times). Australia and the United States have the highest average number of citations per paper, 57.3 and 48.7 times, respectively, indicating that the above countries have significant influence and recognition in this field (**Figure 3C**). From the visualization results of international cooperation, we find cooperation between many countries, among which China and the United States have the closest cooperation (**Figure 4**). Of the institutions that have published the most papers in this field, the United States institutions occupy the top 10 spots, with Harvard University in first place with 167 publications (**Table 3**). The institutions from France, Iran, and China also make the ranking, with increasing annual publications (**Figure 5**). Regarding institutional collaboration, the Fudan University and Nanjing Medical University in China cooperate most closely (**Supplementary Figure 1**).

**Table 2:** The top 10 countries/regions.

Rank	Country	Articles	SCP	MCP	Freq	MCP Ratio	Total citations	Average article citations
1	CHINA	554	488	66	0.3	0.12	8794	15.90
2	USA	347	235	112	0.2	0.32	16902	48.70
3	ITALY	106	86	20	0.1	0.19	4937	46.60
4	GERMANY	95	55	40	0.1	0.42	2845	29.90
5	FRANCE	57	35	22	0	0.39	2739	48.10
6	JAPAN	53	48	5	0	0.09	1561	29.50
7	BRAZIL	51	32	19	0	0.37	881	17.30
8	IRAN	51	28	23	0	0.45	1378	27.00
9	AUSTRALIA	43	24	19	0	0.44	2466	57.30
10	UNITED KINGDOM	41	23	18	0	0.44	1754	42.80

**Table 3:** The top 10 productive institutions.

Rank	Affiliation	Articles	Country
1	HARVARD UNIVERSITY	167	USA
2	UNIVERSITY OF TEXAS SYSTEM	127	USA
3	UTMD ANDERSON CANCER CENTER	103	USA
4	INSTITUT NATIONAL DE LA SANTE ET DE LA RECHERCHE MEDICALE (INSERM)	102	France
5	UNICANCER	89	France
6	UNIVERSITY OF PITTSBURGH	78	USA
7	PENNSYLVANIA COMMONWEALTH SYSTEM OF HIGHER EDUCATION (PCSHE)	66	USA
8	TABRIZ UNIVERSITY OF MEDICAL SCIENCE	66	Iran
9	UNIVERSITE PARIS CITE	65	France
10	SUN YAT SEN UNIVERSITY	57	China

### 3.3 Author and co-cited author analysis

**Figure 6:**
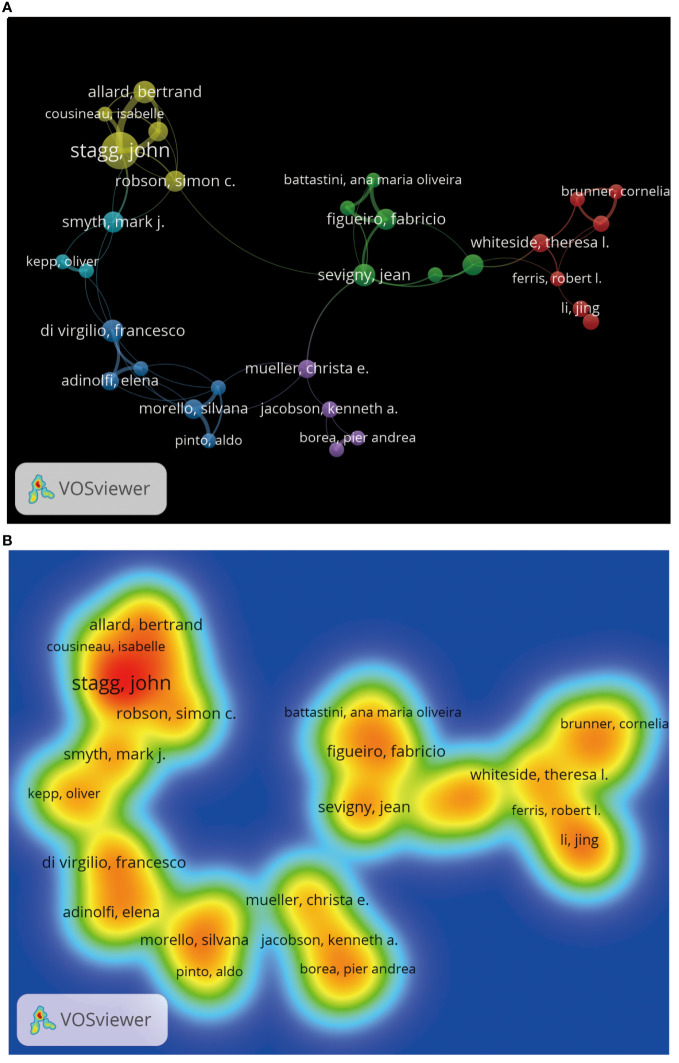
The visualization of cooperation among authors by VOSviewer. **(A)** Diagram of the collaborative network among authors. The same color logo indicates the author cluster group, the thickness of the line reflects the closeness of cooperation, and the node’s size corresponds to the number of papers published by the author. **(B)** Heat map analysis of collaboration among authors. Each label represents an author, and the colors on the heat map are graded from cool to warm, indicating a low to a high degree of cooperation.

**Figure 7:**
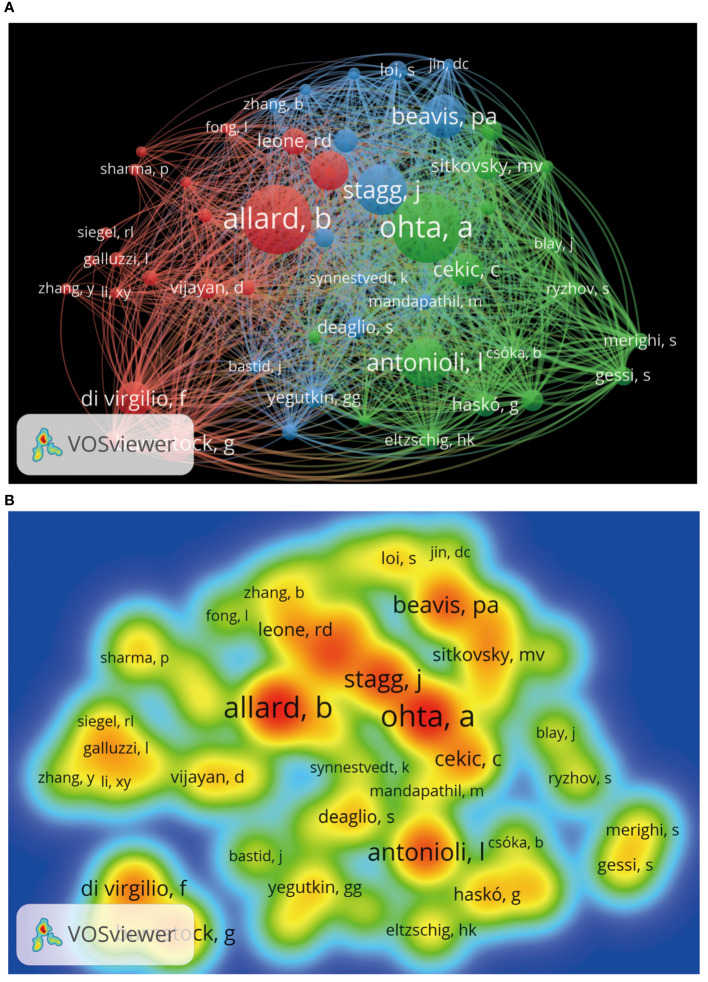
Co-citation analysis of cited authors by VOSviewer. **(A)** Citation analysis of the cited authors. Each node represents an independent author, and the node’s size reflects the number of co-cited papers contributed by the noted author. A larger node means that the author is more co-cited with other authors. Lines indicate co-citation relationships between authors, and the same color represents the same author clustering group. **(B)** Heat map of citation analysis of authors. Each label represents a citation author, the color intensity of the label represents how often the author is cited, and the color gradient on the heat map from cool to warm marks the frequency of co-citation from low to high.

We can understand which authors influence scientific research activities in the adenosine axis and TME through the author’s bibliometrics analysis. A total of 12,065 authors contribute to the publication of papers in this field, of which 23 published at least eight papers. **Figure 6** shows the visual network diagram of the cooperative relationship between the authors of papers in this field. The authors are clustered into six categories, among which the nodes of Stagg J, Smyth MJ, Di Virgilio F, Mueller CE, Figueiro F, and Whiteside TI stand out in each cluster due to their large size, suggesting that they may be relatively active or essential researchers in this field. In addition, the close association between Stagg J and Allard B suggests frequent collaboration between them. **Table 4** shows the top 10 authors with the most published papers, among which Stagg J ranks first with 24 published articles and 2,776 citations, indicating his significant academic influence in this field. The results of the author coupling analysis (**Supplementary Figure 2**) suggest that researchers can be clustered into five categories according to the citation of references. The research directions in the same category are similar, leading them to cite more duplicate references. **Figure 7** shows the co-citation analysis results of reference authors co-cited ≥100 times. Co-cited authors can be clustered into three categories. Stagg J, Allard B, Ohta A, and Antonioli, L occupy a central position in the network and have more lines, indicating that their achievements are cited more than those of other authors and the research of these authors has an essential impact on the field, perhaps because of the high quality of their study, the critical topic of their research, or widespread recognition within the academic community.

**Table 4:** The top 10 most productive and located cited authors.

Rank	Author	Number of documents	H index	Total citations	Average citation per paper	Author	Local citations
1	STAGG J	24	16	2776	116	STAGG J	916
2	LI J	21	10	399	19	ALLARD B	670
3	LIU Y	20	8	257	13	SMYTH MJ	496
4	WANG X	20	9	302	15	YOUNG A	345
5	WANG J	19	10	794	42	ALLARD D	317
6	WANG Y	19	9	233	12	DI VIRGILIO F	273
7	ZHANG Y	19	12	558	29	LOI S	258
8	JADIDI-NIARAGH F	18	14	774	43	ROBSON SC	248
9	LIU L	15	9	629	42	LEONE RD	246
10	ROBSON SC	14	11	1203	86	BEAVIS PA	242

### 3.4 Journals analysis

**Figure 8:**
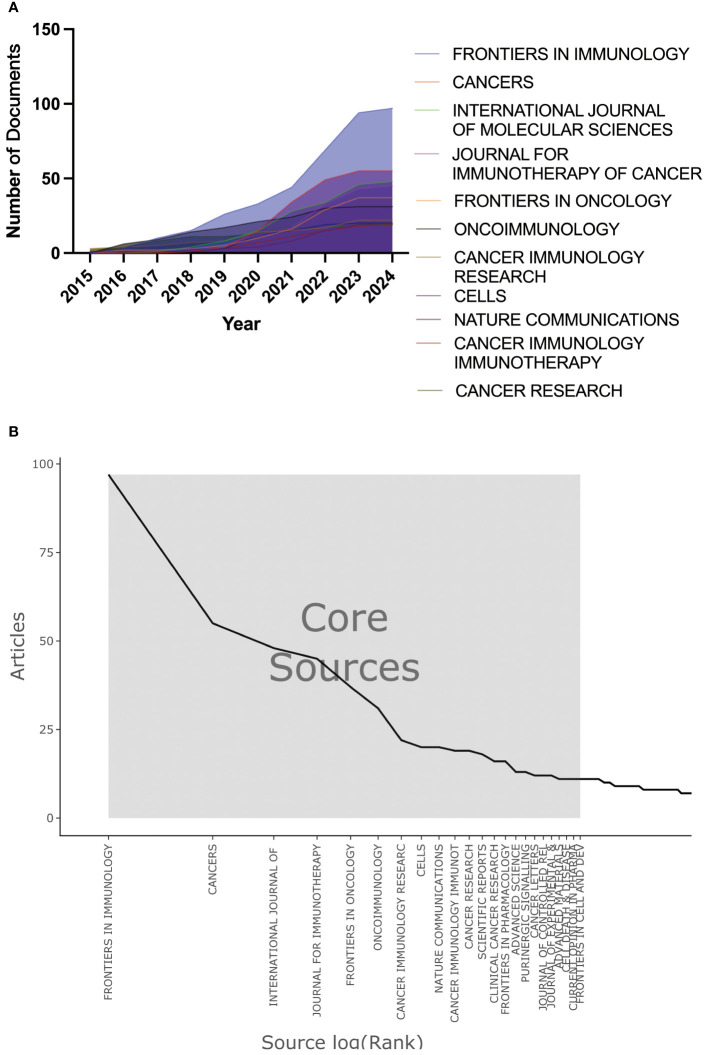
Journal publication trend and distribution map. **(A)** Trend chart of annual publication volume of journals. **(B)** Distribution map of Bradford’s Law for journals.

Through bibliometric analysis of journals, we can identify the academic journals that have played a vital role in the adenosine axis and TME. A total of 579 scholarly journals have published articles in this field. **Table 5** lists the top 10 academic journals with the most published articles. The journal FRONTIERS IN IMMUNOLOGY publishes the most significant number of papers, with 97 articles and a total of 2,317 citations, and the number of publications increased year by year (**Figure 8A**). The journal CANCERS publishes 55 articles with a total of 774 citations. Bradford’s Law helps libraries and researchers identify the most important journals for a particular field. The Bradford’s Law distribution map shows that a few journals constitute the core source of information, which is critical to researchers because they publish most research papers, such as FRONTIERS IN IMMUNOLOGY and CANCERS. (**Figure 8B**). **Supplementary Table 1** presents the details of the top 10 local highly cited journals. **Supplementary Figure 3** shows the co-citation analysis results of co-cited journals cited ≥500 times. CANCER RESEARCH, JOURNAL OF IMMUNOLOGY, and NATURE are the core journals in this field with high co-citation frequency. The close association between CELL, NATURE, and SCIENCE, as well as FRONTIERS IN IMMUNOLOGY and CANCER RESEARCH, shows that they form a core cluster group in the research field, and their research results are often co-cited.

**Table 5:** The top10 productive journals.

Rank	Journal	Number of documents	IF (2023)	JCR (2024)	H index	Total citations
1	FRONTIERS IN IMMUNOLOGY	97	7.3	Q1	25	2317
2	CANCERS	55	5.2	Q1	16	774
3	INTERNATIONAL JOURNAL OF MOLECULAR SCIENCES	48	5.6	Q1	13	856
4	JOURNAL FOR IMMUNOTHERAPY OF CANCER	45	10.9	Q1	17	1228
5	FRONTIERS IN ONCOLOGY	37	4.7	Q2	13	523
6	ONCOIMMUNOLOGY	31	7.2	Q1	20	1190
7	CANCER IMMUNOLOGY RESEARCH	22	10.1	Q1	14	1218
8	CELLS	20	6	Q2	12	500
9	NATURE COMMUNICATIONS	20	16.6	Q1	12	690
10	CANCER IMMUNOLOGY IMMUNOTHERAPY	19	5.8	Q1	11	457

### 3.5 Analysis of cited papers and co-cited references

**Figure 9:**
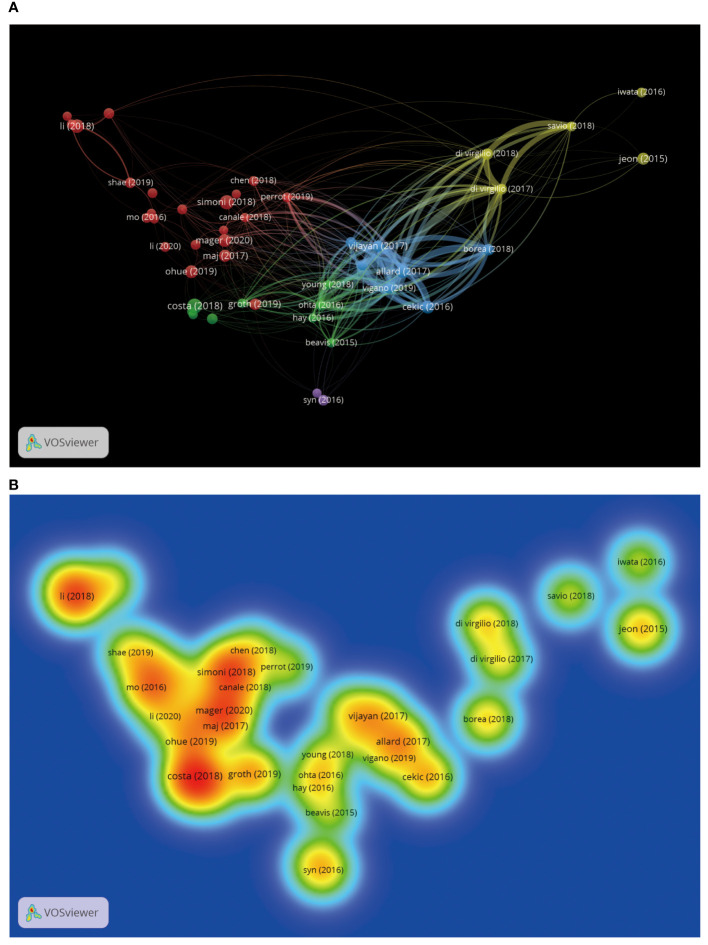
Coupling analysis of research results by VOSviewer. **(A)** Coupling analysis of relevant research results. Each node represents an independent research result, and the size of the node reflects the number of citations contributed by the research result in the bibliographic coupling analysis. The larger the node, the higher the coupling degree between the research result and other research results. The line indicates that there is a coupling relationship between the research results, and the same color represents the same research results cluster groups. **(B)** Heat map of coupling analysis of related research results. Each label represents an independent research result, and the color on the heat map changes gradually from cold to warm colors, marking the degree of coupling of an independent research result from low to high.

**Figure 10:**
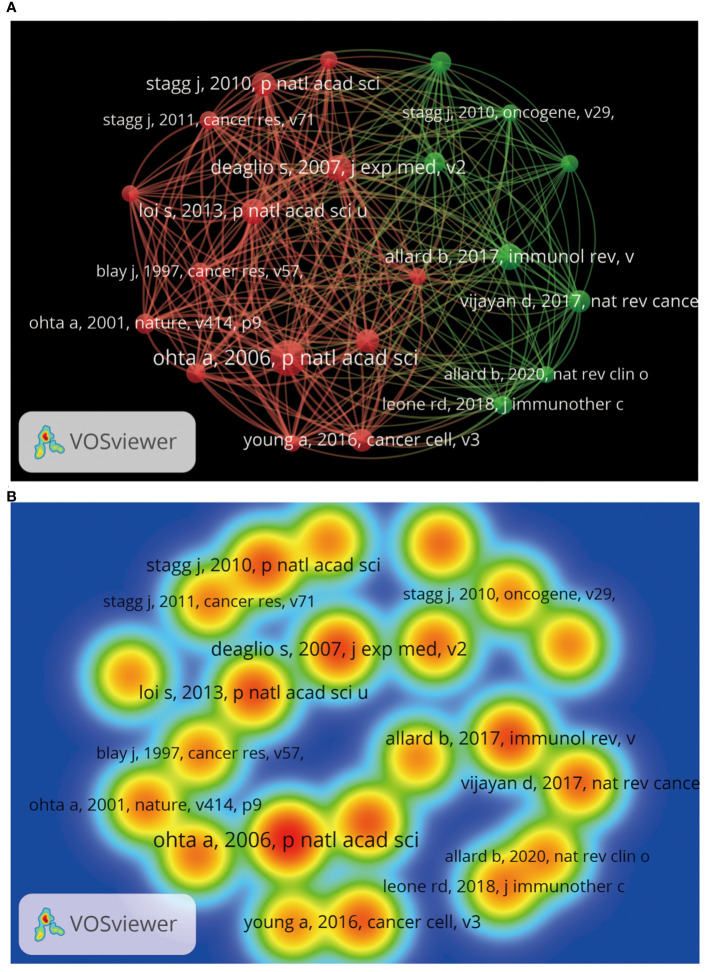
Co-citation analysis of references by VOSviewer. **(A)** Reference co-citation analysis. Each node represents a reference, and the node’s size reflects the number of co-citations of the reference. A larger node means that the reference has a higher co-citation degree with other references. Lines indicate co-citation relationships between references, and the same colors represent the same co-citation reference cluster groups. **(B)** Heat map of reference co-citation analysis. Each label represents a reference, the color intensity of the label represents the frequency of reference co-citation, and the color on the heat map changes gradually from cool to warm colors, marking the frequency of co-citation from low to high.

Through bibliometric paper analysis, we can identify the core papers that have significantly influenced the adenosine axis in TME research. Through an in-depth analysis of the citation patterns, co-citation relationships, and frequency of occurrence of these critical papers, we can trace the origin and development of the research’s knowledge. 1,721 articles and 74,858 citations in this field currently meet our inclusion criteria. **Table 6** presents the field’s top 10 most cited papers. “The ectonucleotidases CD39 and CD73: Novel checkpoint inhibitor targets”, published in IMMUNOLOGICAL REVIEWS by ALLARD B et al., is the most frequently local cited (163 times). **Figure 9** shows the paper coupling network analysis. Papers in the same cluster indicate that they are strongly related academically, probably because they study similar topics, methods, or theories, and many of the same articles are in the reference. These closely connected papers may constitute a research cluster or sub-field in this field. **Figure 10** shows the co-citation network analysis of references co-cited ≥80 times. **Supplementary Table 2** shows the top 10 highly co-cited references. The “A2A adenosine receptor protects tumors from antitumor T cells” was the most co-cited reference (224 times). In this way, researchers can identify critical papers and research trends in the field, which can be very helpful in determining the research directions.

**Table 6:** The top 10 local cited articles.

Rank	Title	DOI	First author	Journal	Year	Local Citations	Global Citations
1	The ectonucleotidases CD39 and CD73: Novel checkpoint inhibitor targets	10.1111/imr.12528	ALLARD B	IMMUNOLOGICAL REVIEWS	2017	163	573
2	Targeting immunosuppressive adenosine in cancer	10.1038/nrc.2017.86	VIJAYAN D	NATURE REVIEWS CANCER	2017	136	445
3	Targeting adenosine for cancer immunotherapy	10.1186/s40425-018-0360-8	LEONE RD	JOURNAL FOR IMMUNOTHERAPY OF CANCER	2018	118	346
4	Adenosine Receptor 2A Blockade Increases the Efficacy of Anti-PD-1 through Enhanced Antitumor T-cell Responses	10.1158/2326-6066.CIR-14-0211	BEAVIS PA	CANCER IMMUNOLOGY RESEARCH	2015	106	237
5	Targeting CD73 in the tumor microenvironment with MEDI9447	10.1080/2162402X.2016.1208875	HAY CM	ONCOIMMUNOLOGY	2016	106	205
6	The adenosine pathway in immuno-oncology	10.1038/s41571-020-0382-2	ALLARD B	NATURE REVIEWS CLINICAL ONCOLOGY	2020	106	242
7	Blocking Antibodies Targeting the CD39/CD73 Immunosuppressive Pathway Unleash Immune Responses in Combination Cancer Therapies	10.1016/j.celrep.2019.04.091	PERROT I	CELL REPORTS	2019	98	254
8	Extracellular ATP and P2 purinergic signaling in the tumor microenvironment	10.1038/s41568-018-0037-0	DI VIRGILIO F	NATURE REVIEWS CANCER	2018	93	427
9	Adenosine 2A Receptor Blockade as an Immunotherapy for Treatment-Refractory Renal Cell Cancer	10.1158/2159-8290.CD-19-0980	FONG L	CANCER DISCOVERY	2020	85	192
10	A Metabolic Immune Checkpoint: Adenosine in Tumor Microenvironment	10.3389/fimmu.2016.00109	OHTA A	FRONTIERS IN IMMUNOLOGY	2016	82	264

### 3.6 Author keywords analysis

**Figure 11:**
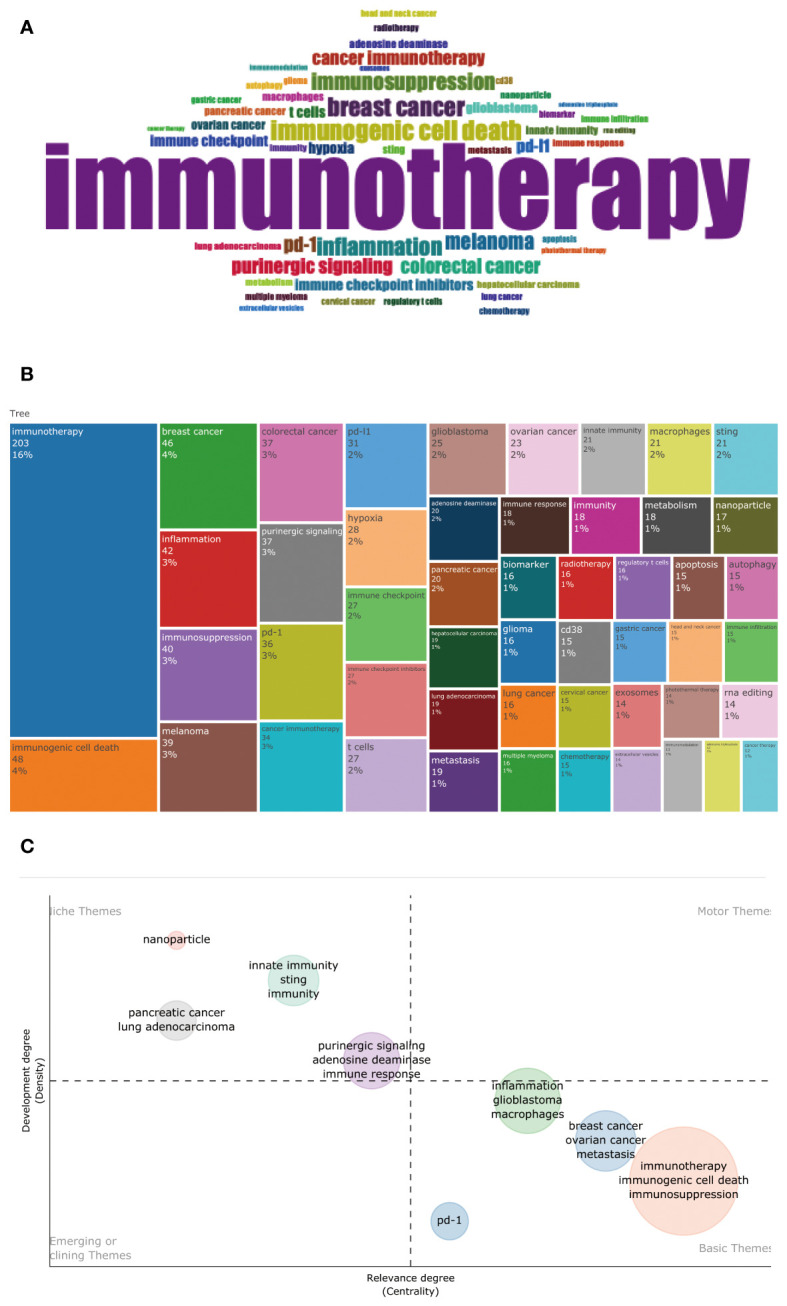
Author keyword analysis. **(A)** Word clouds show author keywords used by authors. The size of an author keyword is directly proportional to its frequency of occurrence; the larger the word, the more frequently it appears in the relevant literature. **(B)** The rectangular dendrogram shows the frequency of keywords with authors. Each different colored rectangle represents an author keyword, and its area indicates how often that author keyword appears in related papers. **(C)** Topic distribution map showing the degree of development and relevance of different research topics in this field. The chart’s vertical axis represents the degree of development of the topic (degree of development), and the horizontal axis represents the centrality or relevance of the topic (degree of relevance).

**Figure 12:**
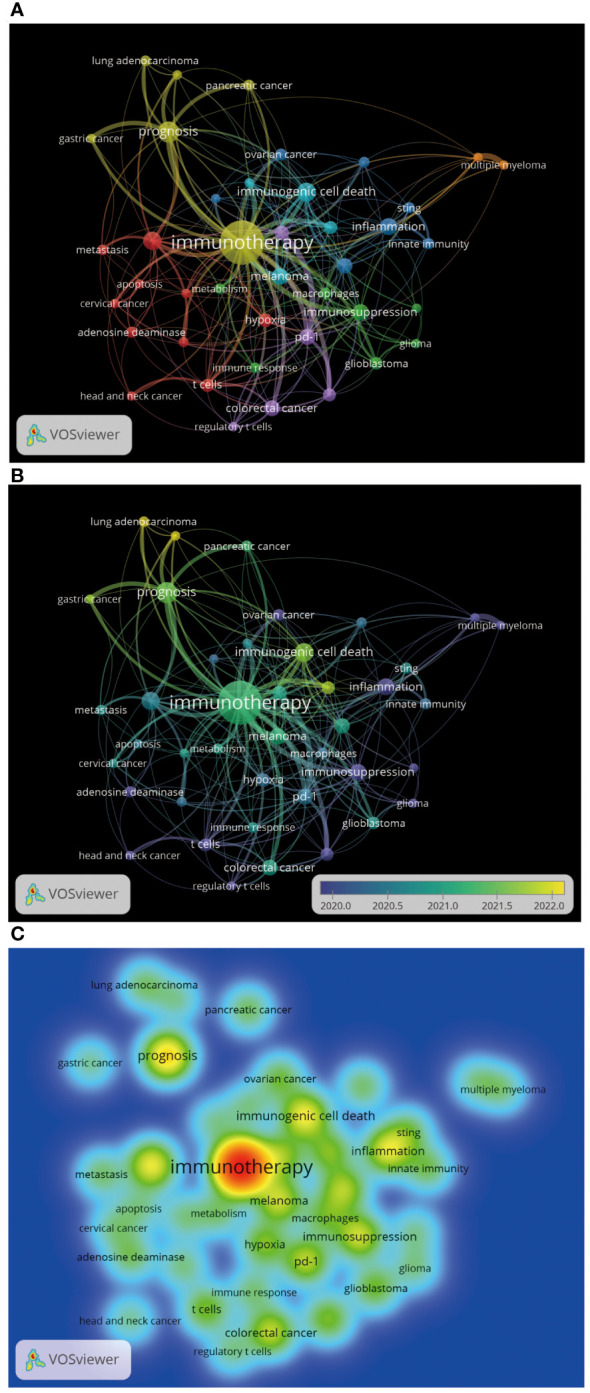
Author keywords co-occurrence analysis by VOSviewer. **(A)** Author keywords co-occurrence analysis. Each node represents an author keyword, and the node size reflects the occurrence frequency of the author keyword. The larger the node, the more frequent the occurrence frequency of the author keyword. The line indicates the co-occurrence relationship between the author keywords, and the same color represents the same author keywords cluster group. **(B)** The time trend chart of author keywords. The color changes gradually from green to yellow, representing the time range from 2020 to 2022. **(C)** Heatmap of author keywords co-occurrence analysis, each label represents an author keyword, and the color intensity of the label represents the occurrence frequency of the author keyword. The color on the heatmap gradually changes from cold color to warm color, indicating that the occurrence frequency of the author keywords changes from low to high.

A total of 3,324 author keywords were included in this analysis **Figure 11A** shows the word cloud of author keywords. It can be seen that immunotherapy occupies a central position in the figure and is shown as the most significant word, indicating that the relationship between the adenosine axis and immunotherapy is a core concept for many researchers in this field. The size of these disease author keywords, such as breast cancer, melanoma, colorectal cancer, lung cancer, ovarian cancer, glioblastoma, pancreatic cancer, and hepatocellular carcinoma, indicates that they are the most frequent cancer types in adenosine-related tumor studies (**Figure 11B**). Immunotherapy, immunosuppression, immune checkpoint, and immune checkpoint inhibitors are the hot keywords in the research, reflecting the importance of the adenosine axis in tumor immunotherapy. Author keywords related to tumor immunity, such as immunogenic cell death, T cells, Sting, regulatory T cells, innate immunity, and immune infiltration, demonstrate the pathways by which the adenosine axis affects the TME. The combination of these author keywords gives a comprehensive view of the importance of the adenosine axis in the research field of the TME, including its role in disease development, treatment response, and prognostic assessment. **Figure 11C** is a topic distribution map, and the author keywords in the adenosine axis and TME field are mainly concentrated in the second and fourth quadrants. Pancreatic cancer, lung cancer, innate immunity, Sting, etc., in the second quadrant, are the core topics with high maturity in current research. In the fourth quadrant, immunotherapy, immunogenic cell death, immunosuppression, macrophages, PD-1, breast cancer, ovarian cancer, and glioblastoma are basic topics with low maturity in the current research field, and these topics may become research hotspots or future development trends. This analysis helps investigators decide which areas to focus on and which emerging areas are worth further exploration. **Figure 12** presents the author keywords co-occurrence analysis with occurrences ≥15 times. It can be seen that the famous author keywords in recent years have been immunotherapy, immunogenic cell death, inflammation, lung cancer, and gastric cancer.

## 4 Discussion

By bibliometric analysis of global annual publication volume, country/region and institution distribution, article authors and co-cited authors, journal distribution of these articles, author keywords and the co-occurrence of author keywords, highly cited articles, and highly co-cited references, we get a comprehensive and in-depth understanding of the research situation of CD39-CD73-eAdo/A2aR in the field of TME. The effect of CD39-CD73-eAdo/A2aR on the infiltration and function of various immune cells in TME, tumor immunotherapy response, and patient prognosis has attracted the attention of researchers from many countries/regions. American scholars still dominate the research in this field, with the highest number of citations and leading in multi-country collaborative publications. Chinese scholars produce the most research results. The journal FRONTIERS IN IMMUNOLOGY has published the wealthiest research in the field, and the number of publications is increasing yearly. Stagg J was a highly influential researcher in this field. Further exploration of targeted inhibition of CD39-CD73-eAdo/A2aR alone or combined with other immunotherapy, radiotherapy, and chemotherapy in treating various cancer types and developing effective clinical therapeutic drugs are continuous research hotspots in this field.

For researchers, it is essential to understand the research hotspots in the field and grasp the research direction. The author keywords analysis module of bibliometrics provides us with the research trends and hot spots related to CD73-eAdo/A2aR in the field of TME. Combined with the keywords analysis results, we summarize the current research hotspots into the following points by reading the latest and high-quality research results in this field.

### 4.1 Adenosine receptors and immunotherapy

Cytotoxic T lymphocytes (CTLs) are antigen-specific effector cells that can eliminate cancer cells contact-dependently. Despite the infiltration of tumor-specific CTLs, metabolic disorder impairs the anti-tumor function of tumor-infiltrating CTLs. Restoring the function of these tumor infiltrating CTLs is vital to improving immunotherapy. eAdo weakened the one-to-one binding of activated effector CTLs to target tumor cells, inhibited the sustained anti-tumor effect, and severely impaired the proliferation ability of CTLs. Antagonism of A2aR signaling stabilized and prolonged CTLs-target cell binding and accelerated the delivery of lethal hits to individual contacts and CTLs (17). The combination of A2aR and TIM3 damaged the cytoskeletal polarization of CTLs *in vivo* and *in vitro* and inhibited CTLs infiltration. Blockade of A2aR and TIM3 may enhance T-cell activation therapies (such as anti-PD-1 and anti-CTLA-4) (18). Hypoxia-induced eAdo also can lead to inhibitory TME and impede the efficacy of ICIs (19). Studies have found that A2aR blockers improved NK cells’ maturation and cytotoxic function and inhibited tumor metastasis in a perforin-dependent manner (20).

In terms of treatment, the combination of adenosine receptor antagonist and anti-PD-1 prolonged the survival of mice with liver cancer (19). Inhibiting PD-1/PD-L1/L2 combined with inhibiting CD73/A2aR can effectively treat diffuse large B-cell lymphoma (21). The A2aR antagonist ciforadenant has shown therapeutic activity and good tolerance in patients receiving immunotherapy for the first time and in patients resistant or refractory to anti-PD-1/L1 treatment in a phase 1 clinical trial in renal cell carcinoma (22). The novel A2aR antagonist DZD2269 could reverse the immunosuppression induced by high concentration eAdo and play a more significant anti-tumor effect when combined with ICIs, radiotherapy, or chemotherapy (23). Taminadenant (PBF509/NIR178) is also a kind of A2aR antagonist that can activate the anti-tumor immune response. Clinical benefit was observed in all patients treated with taminadenant (24).

A2aR is the key immune regulatory factor that protects normal tissues from inflammatory damage and inhibits CTLs from antitumor function of the body. Lack of oxygen and eAdo-rich TME can inhibit the antitumor function of CTLs (25). Respiratory hyperoxia also promoted the regression of spontaneous metastasis from orthotopically grown breast tumors (25). Sitkovsky and collaborators proposed targeting the hypoxia-adenosine-A2aR pathway as a cancer immunotherapy strategy to prevent suppression of antitumor T cells in the TME (13–15).

### 4.2 CD39 and immunotherapy

#### 4.2.1 CD39 and anti-PD-1/L1 immunotherapy

Non-small cell lung cancer patients with EGFR mutations had poor anti-PD-1/L1 efficacy (26). IL-10 induced CD8+T cell immune responses in a CD39-dependent manner, and IL-10 had a limited role in upregulating CD39 expression in EGFR-mutated tumors. Combining recombinant mouse IL-10 protein and PD-1 blocking can optimize the anti-PD-1/L1 therapeutic effect of EGFRL858R mutant lung cancer (26). IPH5201 and IPH5301 antibodies can target human membrane-associated and soluble forms of CD39 and CD73, respectively (27). These antibodies promoted anti-tumor immunity by stimulating DC and TAM and restored the activation of T cells isolated from cancer patients. Cell-specific CD39 expression in macrophages and CD73 expression in liver cancer cells synergistically activated the eATP-eAdo pathway and produced more adenosine, impacting CD8+T cell function and driving anti-PD-1 immunotherapy resistance. Targeting CD39 on macrophages can save liver cancer resistance to PD-1 therapy (28). WU et al. (29) used CD39 inhibitor (CD39i) POM1 and AMP-activated protein kinase agonist metformin, both of which were encapsulated in cancer-cell-derived exosomes and used as nanocarriers for tumor-targeted delivery to achieve metabolic reprogramming of TME, thereby preventing tumor recurrence and overcoming anti-PD-1 resistance. Terminally exhausted T cells are tolerant to immunotherapy. Hypoxia can induce the expression of CD39 on terminally exhausted CD8+T cells. Knocking out CD39 of CD8+ T cells can delay the progress of the tumors, improve immune therapy of reactivity, and enhance the infiltration of tumor-specific T cells (30). Liu et al. (31) found that CD39i can limit bladder cancer growth and improve tumor-burdened mice’s overall survival. ScRNA-seq revealed that CD39i increased the infiltration of NK cells, conventional type 1 dendritic cells, and CD8+T cells and decreased the abundance of Treg cells in the tumor. There was a significant synergy between CD39i and cisplatin, but the CD39i + anti-PD-L1 (or anti-PD1) strategy did not show any synergy in the BC model. The anti-tumor effect of the CD39i strategy combined with anti-PD-L1/PD1 still needs to be further explored.

#### 4.2.2 The value of CD39+CD8+T cells

Monitoring CD39 expression can help quantify bystander T cells. In colorectal and lung cancer, CD39-CD8+ tumor-infiltrating lymphocytes (TILs) generally lack chronic antigenic stimulation and were defined as bystander T cells. CD39+CD8+T cells were characterized by exhaustion, tumor reactivity, and clonal expansion, a feature of chronically stimulated CD8+ TILs (32–36). T-cell receptor (TCR) sequencing results also indicated that CD39+CD8+TILs undergo TCR clonal expansion driven by tumor antigens (34, 37). CD39+CD103+PD-1+CD8+ tumor-resident memory (Trm) T cells could significantly reduce the recurrence risk of patients receiving adjuvant immunotherapy. In the country, patients with relapse had a significantly higher proportion of bystander T cells (CD39-CD103-PD-1-CD8+). According to the spatial distribution analysis of immune cells, CD39+Trm T cells were infiltrated around melanoma cells compared with bystander T cells (38). Trm T cells represented a recurrence-free survival (RFS) biomarker after anti-PD-1 treatment. Higher baseline CD39+CD8+T cell counts improved clinical outcomes in patients with ICBs (36). Moreover, CD39+Trm T cells can recover their antitumor function after ICIs treatment (39). The distribution and regularity of CD39+T cells in non-small cell lung cancer can predict the prognosis of patients (40). Poor 5-year RFS was associated with high CD39 and CD73 expression in tumor stroma. Infiltration of CD39+CD103+CD8+T cells in the tumor nest was associated with better 5-year RFS. The tumor-infiltrating CD39+CD103+CD8+T cells were tumor-reactive immune cells, and the increase of tumor-infiltrating CD103+CD39+CD8+T cells was associated with better overall survival in head and neck cancer (41). CD39 was a significant driver of T-cell depletion in primary and metastatic colorectal tumors. HER-2-specific CD39 disrupted engineered T cells are promising advanced medicinal products for primary and metastatic CRC (42).

In conclusion, CD39 expression on CD8+T cells can be used to identify bystander and Trm CD8+T cells. CD39+CD8+T cells are related to the functional status of CD8+T cells, and their distribution and proportion in the TME are also associated with patients’ sensitivity to immunotherapy, such as anti-PD-1, and their prognosis. Therefore, the identification and dynamic monitoring of CD39 expression in tumor infiltrating CD8+T cells have significant value.

### 4.3 CD73 and immunotherapy

#### 4.3.1 CD73 and anti-PD-1/L1 and anti-CTLA-4 immunotherapy

MEDI9447, a human monoclonal antibody specific to CD73, increased the infiltration of effector CD8+T cells and macrophages in mouse models, alleviated adenosine-mediated lymphocyte inhibition *in vitro*, and inhibited isogenic tumor growth in mice (43). BEAVIS et al. (44) also found that blocking PD-1 could enhance the expression of A2aR on tumor-infiltrating CD8+T cells. Dual blocking of PD-1 and A2aR significantly enhanced the expression of IFN-γ and GzmB in tumor-infiltrating CD8+T cells, thereby enhancing the inhibition of CD73+ tumor growth and prolonging the survival of mice. The combination of AB680, a potent CD73 selective inhibitor, and palbociclib can overcome the adverse reaction of the cyclin-dependent kinase 4/6 inhibitor palbociclib to induce increased expression of PD-L1 in tumors and significantly improve the antitumor efficacy of animal models of colorectal cancer (45). TIGIT/CD155 and CD73 were targeted receptor partners in glioblastoma. The syn notch-engineered pluripotent stem cell-derived NK cells that co-target CD73 and TIGIT/CD155 were effective mediators of anti-glioblastoma response and represented a powerful allogeneic treatment option for this tumor (46).

#### 4.3.2 CD73 and immunotherapy targeting M-MDSCs or CAF

Accumulation of monocytic myeloid-derived suppressor cells (M-MDSCs) also contributed to ICIs resistance. Using mouse models of lung cancer, melanoma, and breast cancer, SARKAR et al. (47) found that CD73+M-MDSCs in TME had a more substantial T cell inhibition function. Tumor-derived PGE2 directly induced M-MDSCs to express CD73 through STAT3 and CREB, which suppressed the effect of ICIs. CAFs constituted the main cell population with high CD73 expression in colorectal cancer. High CAFs abundance and CD73 activity in colorectal cancer were closely associated with poor prognosis (48). From the perspective of studies related to adenosine metabolism and TME, there are still few studies on the effects of CD73+CAF on tumorigenesis and TME, and further studies are needed. In lung cancer clinical specimens and databases, blocking CD73 in the context of induced tumor cell senescence (radiotherapy/chemotherapy) suppressed the tumor and activated anti-tumor immunity, suggesting that targeting CD73 is a novel synergistic anti-tumor strategy in anti-ageing microenvironment (49).

#### 4.3.3 Targeting CD73 in triple-negative breast cancer

Increased CD73 levels were associated with a poor prognosis of immune-cold triple-negative breast cancer. The decrease of CD73 ubiquitination significantly promoted tumor growth and hindered anti-tumor immunity (50). Dual blocking of CD73-TGF-β promoted a variety of inflammatory TME, manifested by decreased levels of MDSCs and M2-like TAM, as well as significantly increased levels of DC activation, cytotoxic T cells, and B cells, accumulation of M1-like TAM, and secretion of TNF-α in triple-negative breast cancer (51). These results provide new strategies for the treatment of triple-negative breast cancer.

#### 4.3.4 Targeting CD73 in pancreatic ductal adenocarcinoma

The surrounding microenvironment of pancreatic ductal adenocarcinoma (PDAC) can significantly promote connective tissue proliferation and immunosuppression. CD73 played a vital role in the pathogenesis and immune escape of PDAC (52). AB680 was used in combination with gemcitabine and anti-PD-1 therapy for the treatment of PDAC in an early clinical trial. AB680 can enhance reactive CD8+T cell infiltration and prolonged mouse survival in subcutaneous and *in situ* mouse PDAC models. Simultaneous administration of AB680 and PD-1 blockers can synergistically inhibit tumor growth (53). The A2aR was a determinant of eAdo-mediated immunosuppression in PDAC (52). Combining anti-CD73 antibodies and A2aR inhibitors can remodel the TME by reducing infiltration of M2-TAM and Treg cells, slowing tumor growth, and reducing metastasis load. In addition, blocking the adenosine pathway may improve the efficacy of combination cytotoxic drugs or immunotherapy (54).

All these studies have indicated that the formation of eAdo promotes the development of immunosuppressive TME in PDAC, which leads to its resistance to traditional and novel therapies. Small-molecule inhibition of CD73 or A2aR can enhance the anti-tumor effect of TME, sensitization immunotherapy, and immunotherapy, providing a novel, efficient, and promising immunotherapy combination strategy for PDAC.

#### 4.3.5 Development of novel CD73 inhibitors

Studies have shown that CD73 is an inhibitory immune checkpoint for various solid tumors. As a result, CD73 antibodies are currently being evaluated for clinical applicability in several multicenter trials. Considering that the efficacy of conventional monospecific CD73 inhibitory antibodies may be limited, PLOEG et al. (55) constructed a new quadrivalent bispecific antibody (bsAb), CD73xEGFR. *In vitro* and *in vivo*, CD73xEGFR targeted the inhibition of CD73/eAdo immune checkpoint and antagonized various cancer-promoting activities of EGFR and CD73. Dalutrafusp alfa is a bifunctional, humanized, glycosylated immunoglobulin G1 κ-antibody that selectively inhibits CD73-eAdo production and neutralizes active TGF-β signaling in patients with advanced solid tumors. Dalutrafusp alfa (45 mg/kg once every two weeks) was well tolerated in patients with advanced solid tumor (56). Biparatopic antibodies with different anticancer activities were prepared from parental anti-CD73 antibodies using a controlled Fab arm exchange technique. Compared with parental antibody, double-antibody sandwich antibody significantly increased the cd73 inhibitory activity, neutralized the inhibitory effect of eAdo on T cell proliferation and IFN-γ secretion, and extended the survival time of tumor-bearing mice (57). Compared with the parent antibody, biparatopic antibodies’ multiple mechanisms of action and more vigorous activity make them promising candidate antibodies targeting CD73 for tumor therapy. This concept could significantly improve future Ab designs.

### 4.4 Adenosine metabolism and radiotherapy resistance

Although radiotherapy (RT) has been highly successful in the treatment of non-small cell lung cancer, local recurrence occurs, and distant effects are rare, even when combined with ICBs. The combination of CD39 inhibitor and RT significantly increased the efficacy of RT. The combination treatment significantly inhibited tumor growth and promoted cytotoxic CD8+T infiltration in the tumor and mobilization of CD8+T cell-dependent antitumor immune response (58). RT can induce immunogenic cell death to activate antitumor immune response. In contrast, activating immune escape processes, such as PD-L1 and the up-regulation of CD39 and CD73, can partially counteract the radiation-induced immune response (59, 60). CHEN et al. (60) constructed nano-scale coordination particle AmGd-NPs by assembling high-Z metal gadolinium (Gd) and small-molecule CD73 inhibitor AmPCP. AmGd-NPs can gradually release AmPCP to inhibit the enzyme activity of CD73, driving a pro-inflammatory tumor microenvironment that promoted DC maturation and enhanced CD8+T cell-dependent antitumor immune response. Ionizing radiation activated the STAT1-IRF1-CD39 axis to up-regulate the expression of CD39. Blocking CD39 enabled eATP accumulation, which activated the dendritic cell NLRP3 inflammasome via the P2X7 receptor, thereby promoting radiation-induced ICD (12). Glioma stem cell ICD vaccine induced by radiation and CD39 inhibition can enhance the expansion of CAR-T in peripheral blood, the multifunction of TME, and the antitumor effect in glioma models (12). Combined blocking of CD73 and PD-L1 can significantly enhance the antitumor effects of Stereotactic body radiotherapy (SBRT) and prolong the survival of patients. Triple therapy (SBRT+anti-CD73+anti-PD-L1) modulated tumor-infiltrating immune cells by increasing IFN-γ+CD8+T cells. In addition, triple therapy reprogrammed cytokines/chemokines in the TME to a more immune-stimulating phenotype (59).

### 4.5 Adenosine metabolism and CAR-T cell immunotherapy

The failure of chimeric antigen receptor (CAR) T-cell immunotherapy to treat solid tumors is partly due to the immunosuppressive microenvironment. During the generation of fully human anti-mesothelin CAR T cells (MSLN-CAR T) (cell activation and transduction), A2aR was significantly upregulated in T cells. Targeting the A2aR signaling pathway may be a promising approach to improve the anti-tumor function of CAR-T cells and may improve clinical treatment outcomes (61). The CRISPR/Cas9 strategy targeted A2aR-edited CAR T cells to fight adenosine-mediated immunosuppression significantly, resulting in enhanced production of cytokines (including IFNγ and TNF) and increased expression of genes associated with the JAK-STAT signaling pathway, thereby improving survival in mice. A2aR-deficient CAR T cells were well tolerated and did not induce significant pathological changes in mice, which supported using CRISPR/Cas9 to target A2aR in the clinic to improve CAR T cell function (62). Liu et al. (63) found that shRNA-mediated A2aR interference can also improve the anti-mSLN-CAR T cell anti-tumor effect *in vivo* and *in vitro*, and proved that shRNA-mediated gene expression modification may be an excellent strategy for improving CAR T cell function in immunosuppressive TME.

### 4.6 Adenosine metabolism and Treg cells

Regulatory T (Treg) cells maintain immune homeostasis by suppressing abnormal/excessive immune responses to self- and non-self-antigens. However, high infiltration of Treg cells is associated with poor survival in various cancers. CD73 can up-regulate CCL5 through the p38-STAT1 axis through the eAdo-A2aR signaling pathway, recruit Treg cells to pancreatic tumors, and cause inhibitory TME (64). Treg cells, which undergo apoptosis due to a weaker NRF2-related antioxidant system and a high vulnerability to free oxygen in TME, can convert large amounts of released eATP to eAdo via CD39 and CD73 to mediate immunosuppression via the eAdo/A2aR pathway. Treg cells in TME can be killed by oxidative stress to maintain and amplify their immunosuppressive capacity (65). CD73+γδTreg cells had more potent immunosuppressive activity than CD4+ or CD8+Tregs (66). Cancer-associated fibroblasts (CAF) derived IL6 induced the differentiation of CD73+γδTreg in normal breast tissue through IL6/STAT3 pathway. CD73+γδTreg, in turn, promoted IL6 secretion by CAFs through eAdo/A2bR/p38MAPK signaling pathway (66). Infiltration of CD73+γδ Treg cells was significantly associated with poor patient prognosis in human breast cancer.

Therefore, there is an urgent need to explore strategies to deplete Treg cells and control their function to increase anti-tumor immune responses in cancer immunotherapy. Targeting adenosine metabolism is an important therapeutic target for Treg cells inhibition.

## 5 Conclusion

The effect of CD39-CD73-eAdo/A2aR on the infiltration and function of various immune cells in TME, tumor immunotherapy response, and patient prognosis has attracted the attention of researchers from many countries/regions. American scholars still dominate the research in this field, but Chinese scholars produce the most research results. The journal FRONTIERS IN IMMUNOLOGY has published the wealthiest research in the field. Stagg J was a highly influential researcher in this field. Further exploration of targeted inhibition of CD39-CD73-eAdo/A2aR alone or combined with other immunotherapy, radiotherapy, and chemotherapy in treating various cancer types and developing effective clinical therapeutic drugs are continuous research hotspots in this field.

## Data Availability

The original contributions presented in the study are included in the article/**Supplementary Material**. Further inquiries can be directed to the corresponding authors.
