# Supplementary material for: Current perspectives and trends of CD39-CD73-eAdo/A2aR research in tumor microenvironment: a bibliometric analysis
Source: Front Immunol. 2024 Aug 12;15:1427380. doi: 10.3389/fimmu.2024.1427380 (PMC11345151; doi:10.3389/fimmu.2024.1427380)
Supplement: Supplementary file 1 [file DataSheet_1.docx]

Supplementary Table 1 The top10 local cited journals.

| **Rank** | **Local cited journal** | **Citations** | **IF (2023)** | **JCR (2024)** |
| --- | --- | --- | --- | --- |
| 1 | CANCER RESEARCH | 3612 | 11.2 | Q1 |
| 2 | JOURNAL OF IMMUNOLOGY | 3032 | 4.4 | Q2 |
| 3 | NATURE | 2813 | 64.8 | Q1 |
| 4 | PROCEEDINGS OF THE NATIONAL ACADEMY OF SCIENCES OF THE UNITED STATES OF AMERICA | 2569 | 11.1 | Q1 |
| 5 | CLINICAL CANCER RESEARCH | 2071 | 11.5 | Q1 |
| 6 | BLOOD | 1975 | 20.3 | Q1 |
| 7 | FRONTIERS IN IMMUNOLOGY | 1884 | 7.3 | Q1 |
| 8 | CELL | 1842 | 64.5 | Q1 |
| 9 | JOURNAL OF BIOLOGICAL CHEMISTRY | 1635 | 4.8 | Q2 |
| 10 | SCIENCE | 1605 | 56.9 | Q1 |

Supplementary Table 2 The top 10 co-cited references.

| **Rank** | **Title** | **DOI** | **First author** | **Journal** | **Year** | **Total Citations** |
| --- | --- | --- | --- | --- | --- | --- |
| 1 | A2A adenosine receptor protects tumors from antitumor T cells | 10.1073/pnas.0605251103 | OHTA A | P NATL ACAD SCI USA | 2006 | 224 |
| 2 | Adenosine generation catalyzed by CD39 and CD73 expressed on regulatory T cells mediates immune suppression | 10.1084/jem.20062512 | DEAGLIO S | J EXP MED | 2007 | 166 |
| 3 | The ectonucleotidases CD39 and CD73: Novel checkpoint inhibitor targets | 10.1111/imr.12528 | ALLARD B | IMMUNOL REV | 2017 | 163 |
| 4 | CD73 promotes anthracycline resistance and poor prognosis in triple negative breast cancer | 10.1073/pnas.1222251110 | LOI S | P NATL ACAD SCI USA | 2013 | 158 |
| 5 | Anti-CD73 antibody therapy inhibits breast tumor growth and metastasis | 10.1073/pnas.0908801107 | STAGG J | P NATL ACAD SCI USA | 2010 | 151 |
| 6 | Targeting CD73 enhances the antitumor activity of anti-PD-1 and anti-CTLA-4 mAbs | 10.1158/1078-0432.ccr-13-0545 | ALLARD B | CLIN CANCER RES | 2013 | 147 |
| 7 | Co-inhibition of CD73 and A2AR Adenosine Signaling Improves Anti-tumor Immune Responses | 10.1016/j.ccell.2016.06.025 | YOUNG A | CANCER CELL | 2016 | 143 |
| 8 | Targeting immunosuppressive adenosine in cancer | 10.1038/nrc.2017.86 | VIJAYAN D | NAT REV CANCER | 2017 | 136 |
| 9 | Immunity, inflammation and cancer: a leading role for adenosine | 10.1038/nrc3613 | ANTONIOLI L | NAT REV CANCER | 2013 | 129 |
| 10 | CD39 and CD73 in immunity and inflammation | 10.1016/j.molmed.2013.03.005 | ANTONIOLI L | TRENDS MOL MED | 2013 | 128 |


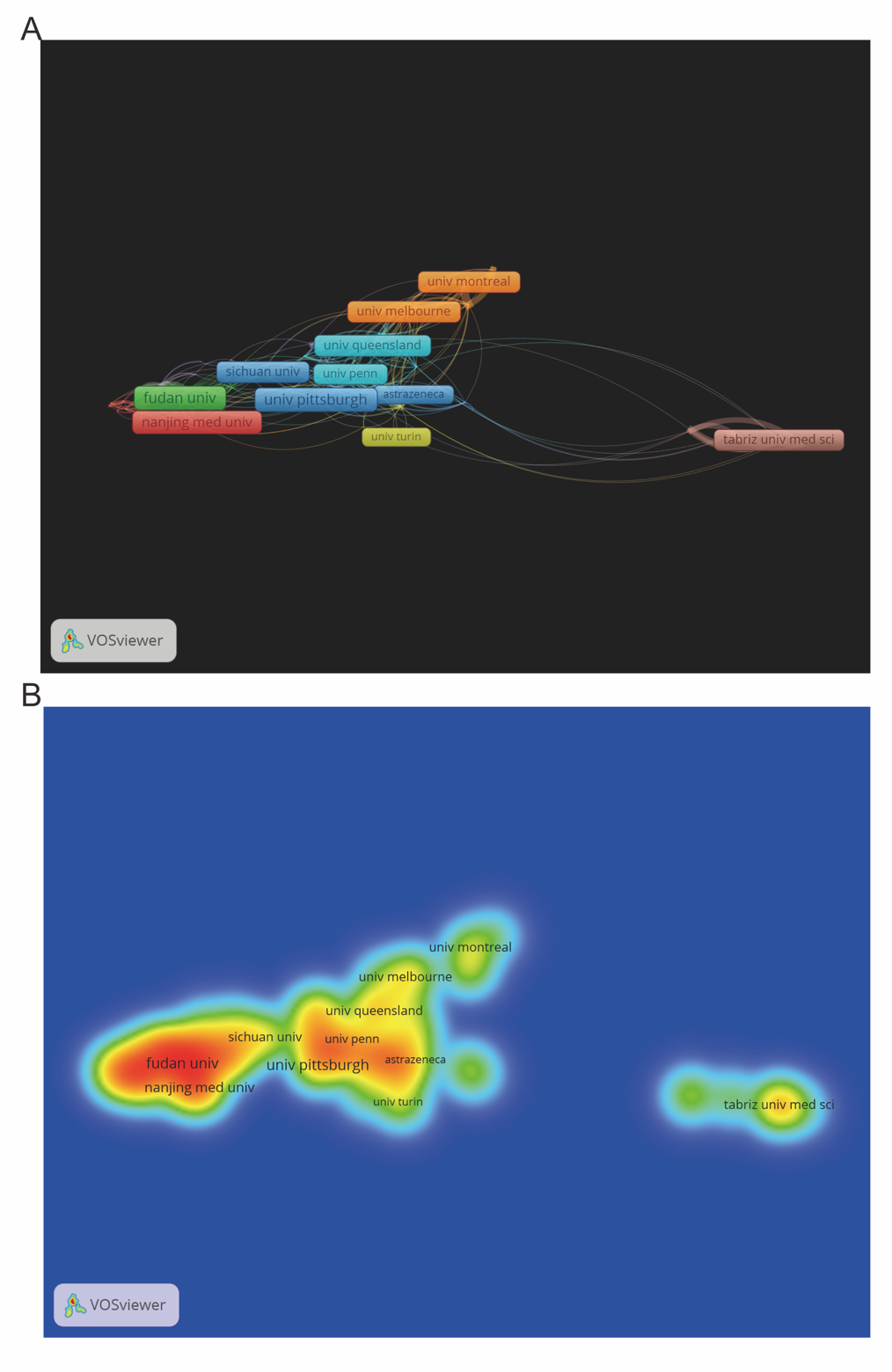


Supplementary Figure 1 Cooperation between institutions. (A) Institutional cooperation network diagram. The same-colored logo represents the same institutional cluster group. The thickness of the line reflects the closeness of cooperation, and the node's size corresponds to the number of papers published by the institution. (B) Heat map of collaboration between institutions. Each label represents an agency. The color on the heat map gradually changes from cool to warm, indicating the degree of cooperation from low to high.


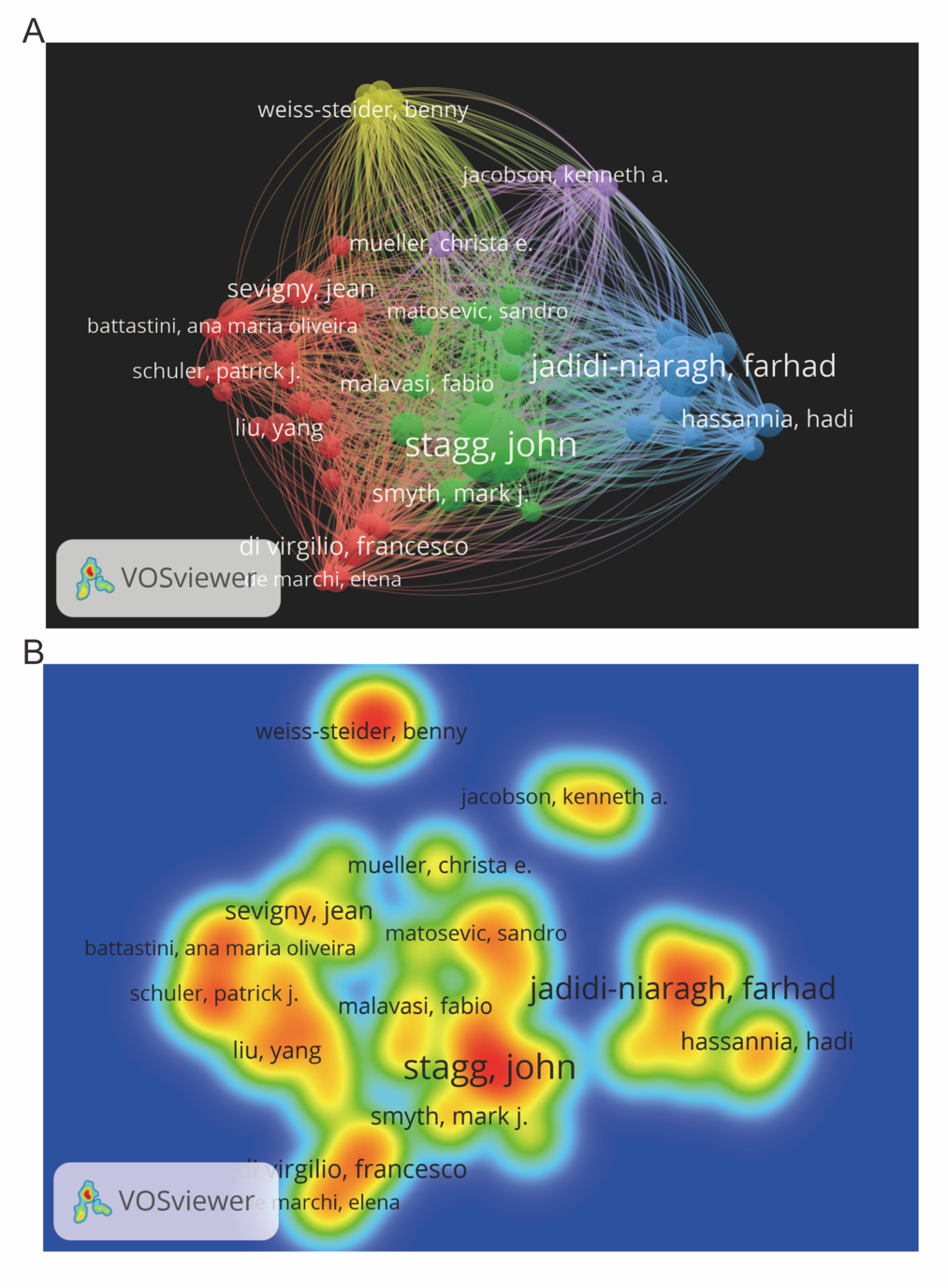


Supplementary Figure 2 Author coupling analysis. (A) Author coupling analysis. Each node represents an independent author, and the node's size reflects the number of papers contributed by the author in the bibliographic coupling analysis. The larger the node, the higher the coupling degree of the author with other authors in the field. The lines indicate the coupling relationship between the authors, and the same color represents the same author cluster group. (B) Heat map of author coupling analysis. Each label represents an author, and the color on the heat map changes gradually from cool to warm colors, marking the degree of coupling of the author from low to high.


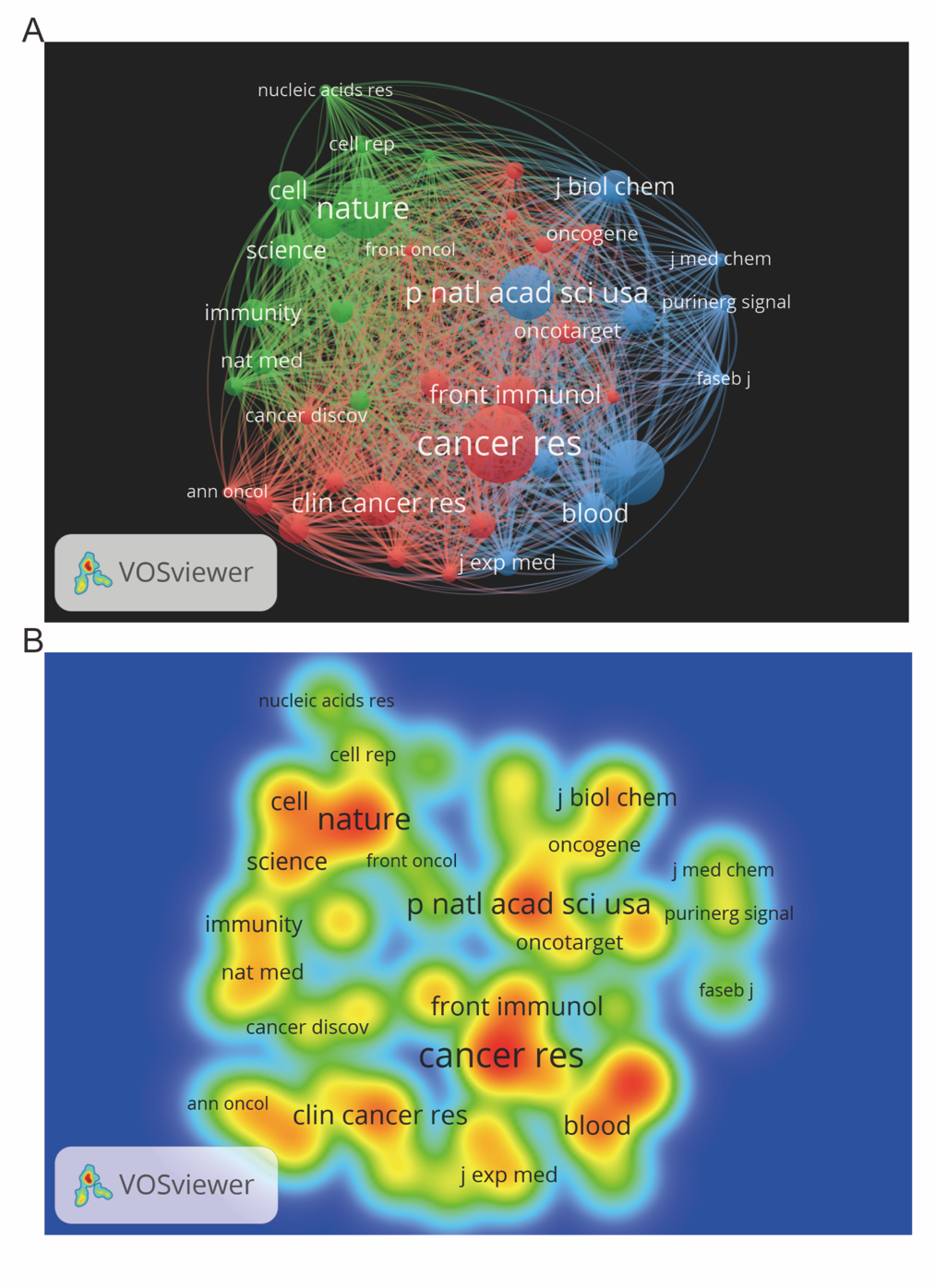


Supplementary Figure 3 Journal co-citation analysis. (A) Journal co-citation analysis. Each node represents a journal, and the node's size reflects the number of co-cited papers contributed by the journal. The larger the node, the higher the co-citation of the journal with other journals in the field. Lines indicate co-citation relationships between journals, and the same colors represent the same journal clustering groups. (B) Heat map of journal co-citation analysis. Each label represents a journal, the color intensity of the label represents the frequency of journal co-citation, and the color on the heat map changes gradually from cold to warm colors, marking the frequency of co-citation from low to high.
